# Immune-Mediated Necrotizing Myopathy With Concurrent Statin Use After Routine COVID-19 Inoculation: A Case Report

**DOI:** 10.7759/cureus.37876

**Published:** 2023-04-20

**Authors:** Zarmina Mufti, Nicholas Dietz, Luke Pearson, Enzo Fortuny, Jersey Mettille, Dale Ding, Martin Brown, Harris Mufti

**Affiliations:** 1 Neurology, University of Louisville, Louisville, USA; 2 Neurosurgery, University of Louisville, Louisville, USA; 3 Neurosurgery, University of Lousiville, Louisville, USA; 4 Anesthesiology and Critical Care, University of Louisville, Louisville, USA; 5 Neurology, University of Louisville, Lousiville, USA; 6 Neurology, Frontier Medical and Dental College, Abbottabad, PAK

**Keywords:** myopathy, immunology, hmcgr antibody, immune mediated necrotizing myopathy, sars-cov-2

## Abstract

SARS-CoV-2 has been associated with multiple disease processes and chronic sequela. Much less understood are the neurological effects, ranging from headaches, pro-thrombotic state, encephalitis, and myopathic processes. Many case reports have documented post-SARS-CoV-2 virus effects; however, this case highlights the possibility of a less commonly described neurological manifestation possibly related to the BNT162b2 mRNA Pfizer vaccine. There is scant literature on immune-mediated necrotizing myopathy (IMNM) triggered after COVID-19 vaccination. The BNT162b2 mRNA COVID-19 vaccine (Pfizer, BioNTech) has proven to be safe and effective in reducing transmission of COVID-19, but post-vaccination neurological events, including venous sinus thrombosis, transverse myelitis, and immune-mediated diseases, such as Guillain-Barré syndrome, have been reported. We report a case of IMNM with HMG-CoA reductase antibody positivity in the setting of BNT162b2 vaccination. The patient presented with progressive muscle weakness with rhabdomyolysis and necrotizing autoimmune myopathy proven on muscle biopsy after the second dose of the BNT162b2 vaccine. Ultimately, this case report highlights the importance of clinical suspicion for early diagnosis and initiation of treatment after symptoms concerning necrotizing myopathy.

## Introduction

Numerous vaccines have made significant strides in medicine, reducing population numbers of infection, morbidity, and death [1,2]. As with most vaccines, rare auto-immune complications have been reported in the literature, such as those seen with Guillain-Barré syndrome and giant cell arteritis related to the influenza vaccine [1,3]. SARS-CoV-2 has been associated with multiple disease processes as well as acute and chronic sequela [4-6]. Much less understood is the extent and pathophysiology of neurological effects, ranging from headaches, pro-thrombotic state, encephalitis, and myopathic processes [7,8]. Many case reports have documented post-SARS-CoV-2 virus effects; however, this case highlights the possibility of a less commonly described neurological manifestation possibly related to the BNT162b2 mRNA vaccine [9,10]. While there are reports of immune-mediated necrotizing myopathy (IMNM) in the setting of COVID-19 vaccination, there is scant literature detailing the breadth of cases and understanding of pathophysiological mechanisms [11,12].

The BNT162b2 mRNA COVID-19 vaccine (Pfizer, BioNTech) has proven to be safe and effective in reducing transmission of COVID-19 with excellent results and minimal side effects [13]. However, post-vaccination neurological events, including venous sinus thrombosis, transverse myelitis, and immune-mediated diseases, such as Guillain-Barré syndrome, have been reported [14]. Additionally, a host of cases regarding inflammatory myopathies have been described, often following a second vaccination dose [15]. We report a case of IMNM with HMG-CoA reductase (HMCGR) antibody positivity in the setting of the second BNT162b2 vaccination. The patient presented with progressive muscle weakness with rhabdomyolysis and necrotizing autoimmune myopathy proven on muscle biopsy after the second dose of the BNT162b2 vaccine. Ultimately, this case report highlights the importance of clinical suspicion for early diagnosis and initiation of treatment after symptoms concerning necrotizing myopathy.

## Case presentation

In July 2021, a previously healthy 68-year-old female presented to the neurology outpatient clinic with approximately three months of worsening proximal muscle weakness concerning a possible myopathic process. The patient’s symptoms began three months prior to presentation, one week after receiving her second BNT162b2 vaccine. Symptoms were first noticed in the lower extremities with difficulty flexing her hip to get into her truck along with difficulty with walking up the stairs. She sustained several falls during this time and gradually began noticing difficulty with proximal arm strength, being unable to reach objects on top shelves, and combing her hair. She also admits to difficulty in swallowing certain solids, mostly pertaining to large pills. She took a stable dose of atorvastatin 40 mg daily for hyperlipidemia for over five years and aspirin 81 mg daily. She had no other contributory medical, social, or family history.

Physical exam

The patient appeared well-groomed and as stated age. The patient’s physical examination was notable for 2/5 strength in deltoids and biceps. Her triceps strength was 3/5 in bilateral upper extremities, and distally her strength was intact: 5/5 in flexor digitorum profundus, extensor carpi radialis longus, interossei, and lumbricals. A 2/5 strength was documented in hip flexion, specifically with 2/5 in the iliopsoas and hip flexors, 3/5 in the semitendinosus and semimembranosus knee flexors, and vastus knee extensors. Distal strength was again full strength in the lower extremities with 5/5 tibialis anterior, gastrocnemius, soleus, and hallucis longus. Sensation to light touch, pain, and temperature was intact in the upper and lower extremities bilaterally. Reflexes were 2+ in the bilateral biceps, triceps, patellar, and Achilles tendons. There was appropriate bulk and tone observed in the upper and lower extremities without any obvious signs of muscle atrophy or wasting. However, gait was effortful and slow, but with a normal stride, and non-antalgic. Her ambulatory symptoms reportedly worsened prior to presentation, and she had been increasingly unable to ambulate without a walker and close assistance.

Laboratory values

On the evaluation of the comprehensive metabolic profile, she was noted to have transaminitis with numbers well above 300 for her aspartate transaminase and alanine transaminase. She was then referred to a gastrointestinal clinician with an eventual non-diagnostic liver biopsy for her elevated transaminases, along with a referral to a rheumatologist with multiple rheumatological labs drawn without conclusion. The patient was ultimately referred to neurology, and at presentation, her transaminases decreased to around 150s and a notably elevated creatine kinase (CK) 9000 IU/L (normal, <190 IU/L).

Hospitalization

**Figure 1:**
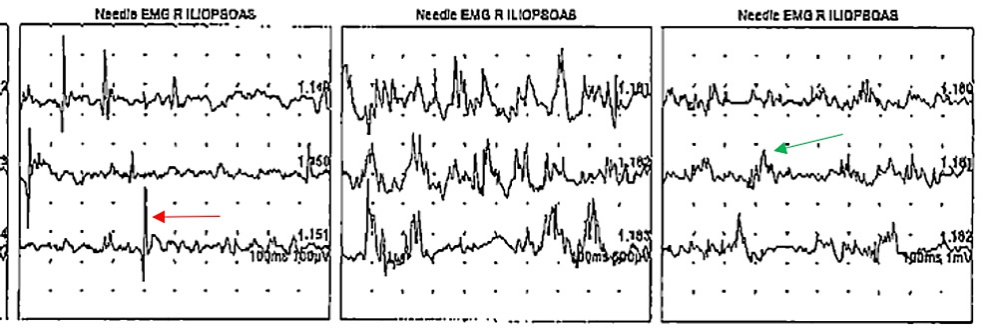
Needle EMG of right iliopsoas muscle showing fibrillation potentials (red arrow) and florid positive sharp waves (green arrow) EMG: electromyogram

The patient was admitted to the neurology inpatient service. During hospitalization, an extensive myositis panel was performed. Specifically, the Quest 8 myositis panel was all negative including EJ, OJ, PL-7, PL-12, Mi-2, Ku, SRP, and Jo-1 antibodies. An electromyogram (EMG) was also done of the quadriceps, deltoids, and triceps that showed short-duration polyphasic motor units with early recruitment in the proximal muscles. In addition, florid positive sharp wave and fibrillation potentials with complex repetitive discharges were most notable in the right iliopsoas and vastus medialis muscles (Figure 1A) and seen to a lesser extent in the right deltoid and triceps.

**Figure 2:**
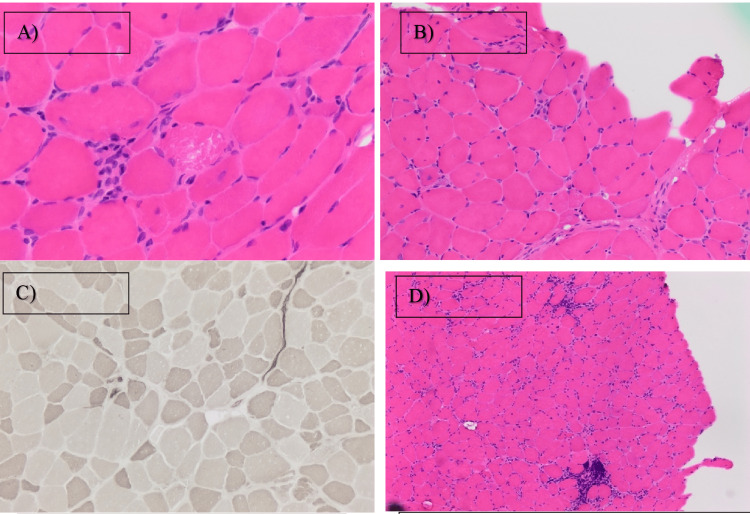
Histopathologic stains of left biceps brachii muscle biopsy (A) Hematoxylin and eosin (H&E) stain 100x magnified showing muscle fiber necrosis, (B) H&E stain 10x magnified showing degenerating/regenerating, atrophic, and necrotic muscle fibers, (C) ATPase 9.4 stain showing variation in fiber size, (D) H&E stain showing patchy, predominantly lymphocytic, endomysial inflammatory cell infiltrate (red circles)

EMG recordings provided sufficient neurophysiologic evidence of an acute myopathic process with neuropathic features. Due to the patient’s CK of >9000 IU and rapidly progressing symptoms, a muscle biopsy was done for precise diagnosis and treatment. A left biceps brachii muscle biopsy was performed that demonstrated an abundance of atrophic and degenerating/regenerating fibers with scattered necrosis associated with patchy, lymphocytic, endomysial inflammation (Figure 2A-C).

These features are consistent with an idiopathic inflammatory myopathy. Toxic/drug-induced myopathy is often characterized by the presence of inflammation restricted to the surroundings of necrotic fibers. This would be consistent with the patient’s use of atorvastatin 40 mg. Moreover, the absence of perifascicular atrophy and perimysial inflammation makes the possibilities of dermatomyositis and anti-synthetase syndrome highly unlikely. From the histopathologic evidence obtained, IMNM was the main differential diagnosis.

After the muscle biopsy, the patient was initiated on 0.5g/kg/day intravenous immune globulin (IVIG) for four days (total of 2g/kg). Her weakness significantly improved after the first dose of IVIG. Her labs were also normalizing: gamma-glutamyl transferase normal and CK down-trending from 9000+ to 6000 IU. Antinuclear antibody was repeated and positive, and c-reactive protein and erythrocyte sedimentation rate were within normal limits. Notably, anti-HMGCR was positive and anti-TIF1 negative. The anti-HMCGR positivity suggests anti-HMGCR myopathy. The patient’s weakness further improved to 4/5 in bilateral upper and lower extremities proximally and was safely discharged with outpatient physical therapy. The patient was counseled to follow up with primary care for malignancy screening.

## Discussion

The present case describes a 68-year-old female with progressive proximal upper and lower extremity weakness and tissue diagnosis suggestive of immune-mediated necrotizing necrosis in the setting of recent inoculation with a second dose of mRNA COVID-19 vaccination and concurrent statin use. Features common to most myopathies are myonecrosis, proximal muscle weakness, elevated CK, and myopathic EMG [16]. However, HMGCR+ with CK levels 50x the upper limit of normal is unique to IMNM [17]. In a recent review of myositis conditions related to COVID-19 vaccination conducted by Syrmou et al., 49 cases were identified [15]. Most cases were observed in females over the age of 55 with the focal case also involving a 67-year-old female who received the second dose of the BNT162b2 mRNA COVID-19 vaccine. Three cases were found to be anti-HMCGR+ and have concurrent statin use. Additionally, 70% of the cases were observed with mRNA-based vaccines.

Underlying muscle damage or exacerbation of pre-existing muscle damage spurred by SARS-CoV2 may have been involved in the presence of myotoxic drugs. The presence of inflammation surrounding necrotic fibers is generally observed in toxic myopathy. Thus, the possibility of a toxic or drug-induced myopathy from a statin drug, for example, is a possible contributing source of this patient's myopathic symptoms. Additionally, the absence of perifascicular atrophy and perimysial inflammation is suggestive of a pathology other than dermatomyositis and anti-synthetase syndrome.

From a pathologic point of view, IMNM was the main differential. A series of 38 patients describe a distinct autoimmune myopathy associated with statin use [18]. Most of these patients were diagnosed with IMNM based on clinicopathologic features such as the ones listed above (absence of perifascicular atrophy, HMGCR+ antibodies, CK levels 50x the upper limit of normal). Another study found similarly that 40% of patients with COVID-19 vaccine-related myositis were on statin [19]. Patients with statin-induced toxic myopathy normally improve after discontinuation of the offending drug such as statins, and patients with IMNM normally have persistent muscle weakness and CK elevation long after discontinuation as seen in this patient. There may be an interaction in which concurrent use of statin may lower the threshold for auto-immune phenomena and synergistically increase the likelihood of IMNM in certain vulnerable populations.

While COVID-19 vaccination has been effective and safe, there are rare autoimmune-related complications associated with administration. Clinicians should be mindful that patients require close follow-up and monitoring and consider the possibility of immunosuppressive management with high-dose steroids or IVIG [15]. EMG and MRI may also assist with diagnosis in identifying muscle edema from IMNM [15]. Further studies may investigate the pathophysiology and mechanism for IMNM related to DNA- or RNA-based vaccination and the role that myotoxic drugs may play synergistically.

## Conclusions

A case of immune-mediated necrotizing myopathy with HMCGR antibody positivity in the setting of the second BNT162b2 dose vaccination was observed in a 68-year-old female with concurrent statin use. Clinical suspicion for early diagnosis and initiation of treatment after symptoms concerning necrotizing myopathy should be considered. Future investigations may explore the pathophysiology of auto-immune phenomena related to COVID-19 vaccination and the role that myotoxic drugs may play synergistically in IMNM.
